# Validating the language mindset inventory in Finland: a study of higher education students’ language-learning mindsets

**DOI:** 10.3389/fpsyg.2025.1655086

**Published:** 2025-08-13

**Authors:** Matthew Billington, Sonja Laine, Kirsi Tirri

**Affiliations:** Department of Education, University of Helsinki, Helsinki, Finland

**Keywords:** mindsets, language mindsets, intelligence, giftedness, validity

## Abstract

**Introduction:**

In recent years, mindset research has increasingly focused on domains of learning, such as mathematics. Foreign/second language (L2) learning is a recent addition to the domain-specific mindset literature. However, few studies have focused on language mindsets in a European context. Moreover, the Language Mindsets Inventory (LMI), the instrument commonly used to measure such mindsets, has not been validated outside North America and Asia.

**Methods:**

To address this gap, the LMI was administered to over 300 students taking compulsory L2 courses at a university in Southern Finland. The construct validity of the LMI was assessed using confirmatory factor analysis (CFA) and hierarchical factor analysis (HFA). The study then used the LMI data to assess the orientation of the students’ language mindsets (fixed or growth) and their correlation with beliefs about general intelligence and giftedness.

**Results:**

The results indicated that the LMI’s three subscales—general language beliefs, L2 beliefs, and age-sensitive beliefs—represent distinct constructs, in turn stratified by growth-mindset (incremental) and fixed-mindset (entity) beliefs. The students’ language mindsets measured by all six resultant factors were more growth oriented than their mindsets about general intelligence and giftedness. In addition, the students’ language mindsets were more growth oriented as measured by the incremental items of the LMI than by the entity (fixed) items.

**Discussion:**

The results suggest that the LMI is a valid instrument for use in Finnish higher education contexts. However, the data do not support combining the scores from the subscales, as the constructs they measure are too distinct. In general, more research is required on why entity and incremental mindset items in mindset scales produce different results about the strength of respondents’ mindset orientations.

## 1 Introduction

In lay speech, we commonly hear that *a head for figures* is required to excel in mathematics. Similarly, we are all familiar with the claim that to learn a foreign language well, an *ear for languages* is necessary. In educational psychology, such beliefs about innate ability are often studied within the framework of mindset theory (e.g., Dweck, 2000).

According to its founder, Carol Dweck, people tend toward either a fixed or growth mindset about human attributes such as intelligence and giftedness (Dweck, 2000). Fixed-mindset individuals view such traits as stable and unchanging, while growth-mindset oriented people consider them malleable and open to development (Dweck, 2012). Among students, fixed mindsets have been found to lead to maladaptive learning responses, such as helplessness (Dweck and Yeager, 2019), the pursuit of performance goals (Dweck et al., 1995), avoidance strategies (e.g., Blackwell et al., 2014) and the belief that effort signifies lack of intelligence (e.g., Blackwell et al., 2014; Dweck, 2012). By contrast, a growth mindset is typically associated with mastery responses (Dweck and Yeager, 2019), learning goals (Dweck et al., 1995), challenge seeking (Dweck and Yeager, 2019), and belief in effort as a key component of learning (Dweck and Leggett, 1988). Consequently, growth mindsets have been linked to positive educational outcomes such as higher academic performance (e.g., Blackwell et al., 2007; although for opposite results in China, see Sun et al., 2021).

In addition to general intelligence, research has increasingly explored innate beliefs about ability in specific domains, positing that individuals can hold different mindsets about different areas of human activity (e.g., O’Keefe et al., 2018). Here, mathematics mindsets have attracted particular attention (e.g., Bostwick et al., 2020 in Australia; Gunderson et al., 2017, in the United States; Heyder et al., 2020 in Germany; Puusepp et al., 2024 in Finland).

Research on language mindsets is a recent addition to the domain-specific mindset literature. In one of the first studies, Mercer and Ryan (2010) investigated beliefs about the role of talent in second language acquisition among higher education students learning English as a second language (L2) in Austria and Japan. They concluded that such mindsets could affect students’ approaches to learning languages. Later, Lou and Noels (2016) exposed a group of Canadian higher education learners to either growth or fixed-mindset ideas about language learning. They found that the former were more likely to endorse learning goals over performance goals and were less likely to exhibit helplessness responses when faced with challenges in language learning. These results were confirmed by Lou and Noels (2017) in a later study measuring language-mindset orientation, also among students at a Canadian university. Subsequent studies by the authors (e.g., Lou et al., 2022) have suggested, for example, that students with a growth language mindset achieve the highest grades in languages.

In further research, a U.S. study by Ozdemir and Papi (2022) found an association between international teaching assistants’ fixed mindsets about language learning and their levels of language anxiety. Moreover, a study in the UK (Lanvers, 2020) found that secondary school students’ language mindsets became more growth-oriented after a mindset intervention. Finally, several studies have explored language mindsets among Iranian university students. For instance, Zarrinabadi et al. (2022a) found that growth language mindsets predicted resilience, while Khajavy et al. (2021) found that fixed mindsets about languages were negatively associated with continued interest in language learning.

Nonetheless, despite the growing interest in language mindsets, little or no peer-reviewed, quantitative studies have investigated language mindsets in continental European settings or within the Nordic countries (for a systematic review of the language-mindsets literature, see Oruç, 2025). Moreover, in Finland, even mindset research on general intelligence is scarce in a higher education context, indicating a significant gap in the literature. More specifically, at present, there is no knowledge about whether higher education students in Finland are more growth-mindset oriented or fixed-mindset oriented about language learning and whether these beliefs differ from their mindsets about general intelligence or giftedness. Furthermore, no knowledge exists about whether these mindsets might vary according to, for instance, gender or language-learning level.

### 1.1 Measuring mindsets

The most popular approach to measuring mindsets is the use of quantitative survey data and, more specifically, various iterations and adaptations of the Implicit Theories of Intelligence Scale developed by Dweck (2000). This scale, according to Combette and Kelemen (2024), has been used by almost 70 percent studies investigating mindsets among adults.

Dweck’s original scale (Dweck, 2000) consists of eight statements. These statements, four reflecting fixed mindset beliefs and four growth mindset beliefs, are rated on a Likert scale from 1 to 6 (commonly, 1 = strongly agree and 6 = strongly disagree). Generally, the fixed items are reversed scored, the items are summed, and a mean score is calculated such that a higher score denotes a more fixed mindset. Using this approach, according to Dweck et al. (1995) and Dweck (2000), respondents with a mean score of 3 or less are growth-mindset oriented and those scoring 4 or more fixed-mindset oriented. Meanwhile, those scoring between 3 and 4 do not adhere to either mindset, or, as other researchers have suggested, they hold a mixed mindset (e.g., Claro et al., 2016).

Dweck’s original scale is still used by many researchers today (Combette and Kelemen, 2024). However, problems with the growth mindset items have been acknowledged, with Levy et al. (1998, p. 1,423) suggesting that respondents might “universally endorse” them due to social desirability bias. Thus, some studies omit the growth items altogether and measure mindsets with the fixed-mindset statements alone. This has been the main approach in Finland (e.g., Jääskö-Santala et al., 2025; Kuusisto et al., 2017; Zhang et al., 2019). Nonetheless, growth mindset items continue to be used by most researchers, including Dweck, who states that measured by a combination of growth and fixed items, around 40 percent of respondents tend to endorse a growth mindset and 40 percent a fixed mindset (Dweck, 2012). In sum, as Combette and Kelemen (2024) observe, considerable variation exists in the way mindsets are measured today, with some utilizing the original scale, others just the fixed items, and others still an abridged version of the 8-item scale consisting of two growth and two fixed items, and some studies relying on a single fixed or growth item. This variation in the measurement of mindsets has been highlighted as problematic for inter-study comparison (Combette and Kelemen, 2024). Moreover, the use of scales comprised of fixed-mindset items alone to measure growth mindsets has been questioned (e.g., Dupeyrat and Mariné, 2005).

According to the traditional view, espoused by Dweck et al. (1995. p. 326), “those who disagree with the entity theory [fixed-mindset] statements … do in fact hold an incremental [growth mindset] theory and do not simply reject the entity theory”. However, Dupeyrat and Mariné (2005) and Grüning et al. (2024) argued that the correlations between fixed and growth mindset items in their data were too low to support this conclusion. Moreover, Grüning et al. (2024) found that female gender predicted a growth mindset as measured by the incremental item in their data while male gender predicted a growth mindset as measured by the entity item. According to the authors, the results suggest that incremental and entity beliefs are distinct but related constructs. However, they list two other possibilities: acquiescence—respondents’ tendency to agree with survey items rather than disagree with them regardless of their content—and poor item wording. A further possibility, as previously mentioned, is social desirability bias (e.g., Combette and Kelemen, 2024).

When measuring domain-specific mindsets, two broad approaches are usually adopted. The first involves modifying the wording of Dweck’s Implicit Theories of Intelligence scale items to refer to the domain in question. For instance, the statement “You have a certain amount of intelligence, and you cannot really do much to change it” might be changed to “You have a certain amount of *math* intelligence, and you cannot really do much to change it” (e.g., Puusepp et al., 2024). The other approach is to develop a new mindset instrument to examine the domain in question, such as in Santos et al. (2022) for chemistry mindsets.

### 1.2 Measuring language mindsets

To date, most studies measuring language mindsets have utilized a new instrument, the Language Mindsets Inventory (LMI), developed by Lou and Noels (2017) (e.g., Collett and Berg, 2020; Khajavy et al., 2021; Zarrinabadi et al., 2022b; for an exception, see Papi et al., 2019). The LMI consists of three subscales, whose content is based on the qualitative findings of Mercer and Ryan (2010) and Ryan and Mercer (2012). These subscales relate to different aspects of a person’s overall beliefs about language intelligence and acquisition—their language mindset. All three subscales contain three fixed-mindset and three growth-mindset statements. The first subscale, general language beliefs, is intended primarily to measure respondents’ beliefs about native language skills. The introduction to these items in the revised version of the survey (Lou and Noels, 2019, p. 541) describes general language intelligence as:

the capacity to use spoken and written language to express what’s on your mind and to understand other people. People with high language intelligence display a facility with words and languages. They are typically good at reading, writing, telling stories, and so on.

The questions themselves mirror the format of Dweck’s general intelligence mindset items, with “language” added to the statement (e.g., “To be honest, you cannot really change your language intelligence”).

The second subscale concerns what the authors term “second language aptitude.” In the revised version of the subscale, the items also resemble the Dweck format (e.g., “no matter who you are, you can always improve your basic ability to learn foreign languages”).

The third subscale relates to beliefs about a so-called “critical period” in language acquisition. The critical period hypothesis (Lenneberg, 1967) proposes that it is extremely difficult or impossible to learn a language to a normal *native-speaker level* after a certain age, usually the onset or end of puberty. The hypothesis is widely accepted in first language acquisition, where it has been substantiated by some rare cases where children exposed to human language only after the onset of puberty and have failed to learn the language to an adult level (Curtiss, 1981). By contrast, the existence of a critical period in second language acquisition remains controversial. A negative correlation between age and L2 acquisition has been found by many studies (e.g., Newport et al., 2001), but the cause—biological, socio-cultural, motivational—remains unclear (see, e.g., Nikolov and Djigunović, 2006).

### 1.3 Validity of the LMI

In their original study, Lou and Noels (2017) investigated the reliability and validity of their new instrument by calculating the internal consistency of the items (Cronbach alphas), examining construct validity with confirmatory factor analysis (CFA) and hierarchical factor analysis, and exploring the scale’s convergent and divergent validity through its correlation with other variables, such as general intelligence mindsets and beliefs about language competence.

They found that the scale items exhibited good internal consistency, but CFA nonetheless indicated that the items did not reflect a single latent construct, language mindsets. The researchers then tested a two-factor model, where the items loaded onto two latent variables— incremental (growth) language mindsets—and a three-factor model, where the items mapped onto three constructs: general language beliefs (GLB), foreign language beliefs (FLB), and age-sensitive beliefs (ASB). None of these models offered a good fit with the data. Finally, the authors tested a six-factor model, where the three factors mirroring the items in the three subscales were divided into fixed (entity) and growth (incremental) beliefs: GLBent, GLBinc, FLBent, FLBinc, ASBent, and ASBinc. This model offered a good model fit.

As the three fixed-mindset factors were more closely correlated with each other than with the growth mindset factors, the authors then sought a more “parsimonious” model through hierarchical factor analysis, postulating that the data might indicate a second-order structure. In other words, higher-order factors underlay the six first-order factors. They tested a one and two higher-order factor structure, with the one-higher-order model showing reasonable model fit and the two higher-order factor structure (entity and incremental mindsets) a good model fit. The authors explain these findings thus:

Importantly, beliefs about language ability in general (pertaining largely to the native language) were distinct from beliefs about L2 learning, and these two sets of beliefs were distinct from beliefs about maturational constraints. Nonetheless, these three kinds of beliefs can be further differentiated in terms of incremental and entity mindsets, and it is this second dimension of mindsets that provides a broader umbrella for understanding language aptitude beliefs (Lou and Noels, 2017, p. 229).

On this basis, the authors propose that the LMI can be used in three ways. First, the scores from the six factors remain separate; second, two composite scores are created, one from the fixed items of the three scales and one from the growth items of the three scales. Finally, they suggest the creation of a single composite score from all the items for comparison with variables involving complex analyses. However, they warn that when using such a composite score, “nuances can be missed” (2017, p. 229). In terms of future studies, the authors emphasize that more research is needed to confirm the validity of the scale outside North America, and, since the revision of the scale items in 2018, that “more psychometric research is needed” (Lou and Noels, 2019, p. 542) on the scale in general.

Such further psychometric testing of the LMI has remained limited, confined largely to studies with Iranian university students. For instance, Khajavy et al. (2021) used CFA to test a one and two-factor first-order model of the LMI. They concluded that a two-factor model (language entity beliefs and language incremental beliefs) offered the best fit after removing three of the 18 items because of low factor loadings. However, they did not perform hierarchical factor analysis, nor did they report the goodness of fit statistics for the CFA on the LMI in their article. In turn, Zarrinabadi et al. (2022b) tested a two-factor model of the LMI, concluding that it offered a good model fit: (*χ*^2^ = 215.691, df = 128, *χ*^2^/df = 1.685, CFI = 0.927, SRMR = 0.084, RMSEA = 0.057). However, they did not state whether they tested any other models. Moreover, they failed to specify whether the model was a first-order or hierarchical model. In addition, in a study on resilience that included Nigel Luo as a coauthor, Zarrinabadi et al. (2022a) used just one subscale of the LMI. CFA indicated that this subscale did not reflect one underlying construct, foreign language mindsets; rather, it mapped onto two latent variables: fixed mindsets and growth mindsets about foreign language learning. The authors then concluded that the scores for the fixed and growth mindset items should not be combined for comparison with other variables. Finally, outside Iran and North America, Collett and Berg (2020) tested a translated version of the LMI in Japan, finding that none of the CFA models mentioned above fit the data adequately in their sample. Instead, they proposed a four-factor structure consisting of two fixed-mindset variables representing lack of agency and age-related restrictions to language learning and two growth-mindset variables reflecting the potential to increase ability through effort and a more general orientation toward the malleability of language ability.

In sum, to the authors’ knowledge, no studies have tested the validity and parametric characteristics of the LMI outside North America and Asia. Moreover, the relative novelty of the instrument, the different approaches used to assess its factor structure, and the somewhat contradictory findings on that structure point to a clear need for further research.

### 1.4 The present study

The present study aims to fill the two gaps in the research literature described above by attempting to validate the LMI in a novel context—Finnish higher education—and then using the instrument to assess the innate beliefs of university students in Finland about second language learning. More specifically, the study seeks to answer the following research questions:

*RQ1*: What are the psychometric properties of the Language Mindset Inventory in a Finnish higher education setting?

*RQ2*: What mindsets do university students in Finland hold about learning a second language?

## 2 Method

### 2.1 Procedure and participants

The study focused on students studying a second language at the language center of a Finnish university in Southern Finland. In the Finnish university system, students must obtain mandatory credits in a second language as part of their degree program, irrespective of their major. The courses providing these credits are generally offered by so-called *language centers*—independent organizations within the university—rather than by departments of modern languages, which cater to students majoring or minoring in a particular language. Language center students can thus be considered a representative cross-section of the university population and therefore more apt as study participants than students who have specifically chosen to study languages.

Permission was sought and received from the head of the language center to collect data from the students. Ethical approval was also requested from and granted by the university, where the first author works as a language teacher. The study was part of a larger longitudinal research project: CoPErNicus—Changing Mindsets about Learning: Connecting Psychological, Educational, and Neuroscientific Evidence. The project employs a multidisciplinary approach, integrating psychological, educational, and neuroscientific data to examine the views of students, teachers, and parents on learning. The project has received prior ethical approval from the University of Helsinki Ethical Review Board for mindset-focused research conducted in their studies.

Next, in early 2024, teachers at the language center were contacted by email and asked to pass on to their students the link to an electronic survey containing the LMI and the four entity-theory items from Dweck’s eight-item Implicit Theories of Intelligence Scale (ITI) (Dweck, 2000). The survey also included an implicit theories of giftedness scale (ITG) formed from the same four entity items with the word “intelligence” replaced by the word “giftedness” (the approach used in, e.g., Kuusisto et al., 2017; Zhang et al., 2020). The electronic survey, which was created using the online survey tool Qualtrics, also collected background information on the students, including gender, age, nationality, language level of the language course, and the language studied.

Because of the low initial number of responses to the survey, in August 2024, the first author contacted teachers at the language center again and sought permission to visit their lessons to ask the students to complete the survey during class. Eventually, 388 students responded.

After collection, the data were then exported to SPSS, and responses from students who had failed to complete both the LMI and the Implicit Theories of Intelligence Scale and the giftedness scale in full were removed. After this cleaning process, the data comprised responses from 304 students (see Table 1).

**Table 1:** Background variables.

Measure	Category	Frequency	Number
Gender *N* = 304	Female	54.3%	165
	Male	44.1%	134
	Non-binary/no info	1.6%	5
Nationality *N* = 304	Finn	61.8%	188
	Other	38.2%	116
University *N* = 304	1	98.4%	299
	Other*	1.6%	5
Language studied *N* = 304	English	43.4%	132
	Swedish	7.9%	24
	Finnish	10.9%	33
	Spanish	15.8%	48
	French	11.5%	35
	Portuguese	4.6%	14
	Russian	4.9%	15
	Other	1.0%	3
Language level *N* = 283	A1	22.0%	67
	A2	14.5%	44
	B1	16.1%	49
	B2	8.6%	26
	C1	29.6%	90
	C2	2.3%	7

*These students were not majoring at the university but were taking university language center courses.

Of these students, the majority described themselves as Finns or Finnish dual nationals (61.8%). The remainder of the respondents were drawn from 45 countries, with the largest number of responses from Vietnamese students (6.9%), Chinese students (4.9%) and Russian students (1.9%). Of the respondents, 165 identified as male, 134 as female, and four as non-binary. One respondent declined to say. In turn, the students’ age ranged from 17 to 74 years, with a mean age of 24 years.

Of the languages studied, English accounted for the largest proportion of respondents (43.4%), followed by Spanish (15.8%) and French (11.5%) from a total of eight languages. The level of the courses ranged from CEFR A1–C2, with the largest number of courses at the C1 level (31.8%) followed by A1 (23.7%).

### 2.2 Data analysis

The factor structure of the LMI was then examined using confirmatory factor analysis (CFA) and hierarchical factor analysis (HFA) in SPSS Amos. In addition, CFA was performed on the general intelligence and giftedness scales. Then, various parametric tests (Pearson’s *r*, independent samples *t*-tests, paired samples *t*-tests and one-way ANOVAs) were conducted on the data to examine the associations between the LMI subscales, their relationship to the general intelligence and giftedness scales, and their associations with various background variables, such as age, gender, nationality, and course level.

## 3 Results

### 3.1 RQ1: what are the psychometric properties of the language mindset inventory in a Finnish higher education setting?

First, the construct validity of the LMI was investigated using the same four CFA models tested in Lou and Noels (2017). The one-factor model posited language mindsets as the unidimensional latent factor, while the two-factor model hypothesized entity and incremental language mindsets as the two latent factors. In turn, the three-factor model tested general language beliefs, foreign language beliefs, and age-sensitive beliefs as the three underlying factors. Finally, the six-factor model used general language entity beliefs, general language incremental beliefs, foreign language entity beliefs, foreign language incremental beliefs, age-sensitive entity beliefs, and age-sensitive incremental beliefs as the latent factors.

Similar to the results of Lou and Noels (2017), the data showed a poor fit for the first three models, but with one difference: whereas, in Lou and Noels (2017), the two-factor model offered the best (though still poor) fit of these three models, in the present study, the best of these poor-fitting models was the three-factor solution. In contrast to these poor-fitting models, and in line with Lou and Noels (2017), the six-factor model offered a good fit with the data (see Table 2).

**Table 2:** Goodness of fit statistics from confirmatory factor analysis of the LMI.

Model	*χ* ^2^	df	*χ*^2^/df	RMSEA	CFI	TLI
1 factor	1024.163	135	7.586	0.147	0.718	0.681
2 factor	718.597	134	5.363	0.120	0.815	0.789
3 factor	671.174	132	5.085	0.116	0.829	0.802
6 factor	181.705	120	1.514	0.041	0.980	0.975

Then, following Lou and Noels (2017), three higher-order models were tested using hierarchical factor analysis (HFI). These models were a single higher-order factor model (mindsets), a two higher-order-factor model (entity mindsets and incremental mindsets) and a three higher-order-factor model (general language beliefs, foreign-language beliefs, and age-sensitive beliefs). Similar to the findings of Lou and Noels, the one-factor second-order model offered the worst (but still acceptable) model fit for the Finnish data, while the two-factor second-order model offered the best fit (Table 3). Nonetheless, all three models fit the data worse than the six-factor CFA model.

**Table 3:** Model fit statistics from hierarchical factor analysis on the LMI.

Model	*χ* ^2^	df	*χ*^2^/df	RMSEA	CFI	TLI
1 factor	332.807	129	2.580	0.072	0.935	0.923
2 factor	292.869	128	2.288	0.065	0.948	0.935
3 factor	300.585	126	2.386	0.068	0.945	0.933

As the six-factor first-order model produced the best model fit, the parametric properties of these factors were then examined more closely. First, their internal consistency was investigated by calculating Cronbach’s alpha. The results showed that the internal consistency of most factors was good, while it was excellent for general language entity beliefs and acceptable for age-sensitive entity beliefs (Table 4).

**Table 4:** Internal consistency of the six factors of the LMI.

Factor	Cronbach’s *α* (*N* = 304)
GLB-ent	*α* = 0.904
GLB-inc	*α* = 0.830
L2B-ent	*α* = 0.844
L2B-inc	*α* = 0.844
ASB-ent	*α* = 0.735
ASB-inc	*α* = 0.835

Next, the correlations between the six factors were examined more closely (Table 5). The results showed that all the correlations were highly statistically significant (*p* = <0.01). In terms of the strength of the correlation, most factors correlated moderately with each other. However, a weak negative correlation was found (*r* = <0.4) between age-sensitive entity (fixed-mindset) beliefs and general language incremental (growth-mindset) beliefs. Similarly, age-sensitive entity beliefs and second-language incremental beliefs were weakly negatively associated.

**Table 5:** Correlations between the six LMI factors and the ITI and ITG.

N 304	GLBinc	GLBent	L2Binc	L2Bent	ASBinc	ASBent
*GLBinc*	1	−0.560	0.609	***−0.423***	0.574	***−0.295***
*GLBent*	−0.560	1	−0.468	**0.665**	***−0.533***	0.494
*L2Binc*	**0.609**	***−0.468***	1	−0.596	**0.626**	−0.315
*L2Bent*	−0.423	**0.665**	−0.596	1	−0.559	0.474
*ASBinc*	0.574	−0.533	0.**626**	−0.559	1	**−0.602**
*ASBent*	***−0.295***	0.494	***−0.315***	0.474	−0.602	1

All correlations were significant at the <0.01 level (two-tailed). The strongest correlations in each column are in bold, the weakest in italics and bold. In psychology, *r* values of <4 = weak correlation, 4–<7 = moderate correlation, 7–<10 = strong correlation (Akoglu, 2018).

In addition, factors measuring entity beliefs were often more closely correlated with each other than with factors measuring incremental beliefs. For example, the strength of the positive correlation between general language incremental beliefs and second language incremental beliefs was *r* = 0.602. By contrast, the negative correlation between general language incremental beliefs and second-language entity beliefs was *r* = −0.423.

Furthermore, within each of the three subscales of the LMI, correlations between the incremental and entity items were only moderate. Moreover, in some cases, the correlations between the factors *within* the subscales were weaker than the correlations *between* them. For example, second language entity beliefs were more strongly correlated with general language entity beliefs than with second language incremental beliefs.

Then, the convergent and divergent validity of the LMI was assessed by examining the correlations between the six factors of the LMI and Dweck’s Implicit Theories of Intelligence Scale and the Implicit Theories of Giftedness Scale. Prior to this, CFA was performed on these scales to assess whether they could be combined (i.e., whether they reflected a single underlying factor). The results of the CFA (see Table 6) indicated that the scales measured two distinct constructs. Therefore, a composite score was not used in the subsequent analysis.

**Table 6:** CFA on the implicit theories of intelligence and implicit theories of giftedness scale.

Model	*χ* ^2^	df	*χ*^2^/df	RMSEA	CFI	TLI
1 factor	760.559	20	38.028	0.338	0.710	0.594
2 factor	39.186	19	2.062	0.059	0.991	0.987

As can be seen from Tables 5–7, all six factors of the LMI were more strongly correlated with each other than with the Implicit Theories of Giftedness scale, indicating divergent validity. However, the results were more ambiguous for the Implicit Theories of Intelligence scale.

**Table 7:** Correlations between the ITI and ITG and the six LMI factors.

N 304	GLBinc	GLBent	L2Binc	L2Bent	ASBinc	ASBent	ITI	ITG
*ITI*	−0.359	**0.623**	−0.359	0.579	−0.406	***0.350***	1	0.549
*ITG*	*−0.255*	*0.456*	*−0.263*	*0.421*	*−0.257*	***0.253***	**0.549**	1

All correlations were significant at the <0.01 level (two-tailed). The strongest correlations in each column are in bold, the weakest in italics and bold. In psychology, *r* < 4 = weak correlation, 4–<7 = moderate correlation, 7–<10 = strong correlation (Akoglu, 2018).

For instance, general language entity beliefs were more strongly associated with the ITI scale (*r* = 0.623) than they were with *any* of the other LMI factors apart from L2 entity beliefs (*r* = 0.665). Furthermore, second-language entity beliefs were more strongly correlated with the ITI than they were age-sensitive entity beliefs, age-sensitive incremental beliefs, and general-language incremental beliefs. Finally, the association between age-sensitive entity beliefs and the general and second language belief factors (*r* = −0.295, 494, −0.315, 0.474) was weaker than the correlation between the ITI scale and those factors (*r* = −359, 0.623, −0.359, 0.579).

In turn, the Implicit Theories of Intelligence scale correlated moderately with the Implicit Theories of Giftedness Scale (*r* = 0.549). Interestingly, however, it was more strongly associated with general language entity beliefs (*r* = 0.623) and second language entity beliefs (*r* = 0.579) than with the items of that scale.

### 3.2 RQ2: what mindsets do university students in Finland hold about learning a second language?

Based on the results of the CFA and HFA, the study participants’ language mindsets were assessed using the six-factor solution. First, the means of the fixed and growth items of the three subscales of the LMI were calculated alongside the means of the Implicit Theories of Intelligence scale (ITI) and the Implicit Theories of Giftedness scale (ITG) (Table 8).

**Table 8:** Means and standard deviations of the six LMI factors and the ITI and ITG scales.

Factor/scale name *N* = 304	Mean	SD
General language beliefs: entity*	2.648	1.149
General language beliefs: incremental	2.257	0.895
L2 beliefs: entity*	2.610	1.021
L2 beliefs: incremental	2.223	0.840
Age sensitive beliefs: entity*	2.476	0.994
Age sensitive beliefs: incremental	2.358	0.936
Implicit theories of* intelligence	2.890	1.294
Implicit theories of giftedness*	3.570	1.228

*Items were reverse scored for easier comparison. Scores of 3 and under = growth mindset, scores of 4 and above = fixed mindset (Dweck et al., 1995; Dweck, 2000).

On average, the participants held a growth mindset about languages as measured by all six factors of the LMI. Moreover, on average, the participants were more growth oriented about languages than about general intelligence and giftedness. A paired samples *t*-test showed that this difference was statistically significant at the *p* < 0.001 level between all six factors of the LMI and the ITI, with *t*-values ranging from *t*(303) = 3.946 to *t*(303) = 9.198. This was also the case for the difference between the six LMI factors and the ITG, with even larger *t*-values: *t*(303) = 13.707 to *t*(303) = 18.180.

Furthermore, the reversed mean scores for the fixed mindset (entity) items were higher than the mean scores for growth-mindset (incremental) items in all three subscales. In other words, the participants were more growth-minded as measured by the incremental items than by the entity items. A paired-samples *t*-test confirmed that this difference was statistically significant: GLB, *t*(303) = 6.937, *p* < 0.001; L2B: *t*(303) = 7.918, *p* < 0.001; ASB: *t*(303) = 2.394, *p* < 0.05.

In terms of the proportion of students who held a growth, fixed, or mixed mindset about languages, roughly three-quarters were growth minded as measured by the fixed mindset items of the three subscales, while this figure was even higher as measured by the growth mindset items (Table 9). In turn, between 10 and 16 percent of students were fixed-mindset oriented as measured by the fixed-mindset items, while this figure fell to 4–6 percent when measured by the growth mindset items. Finally, between 9 and 13 percent of students held a mixed mindset about language learning as measured by the fixed-mindset items, and between 9 and 12 percent as measured by the growth-mindset items of the LMI.

**Table 9:** Number and proportion of students holding a growth, fixed, or mixed mindset by subscale.

Mindset	GLB-ent	GLB-inc	L2B-ent	L2B-inc	ASB-ent	ASB-inc
Fixed	16% (*n* = 49)	6% (*n* = 19)	12% (*n* = 36)	4% (*n* = 11)	10% (*n* = 29)	6% (*n* = 19)
Mixed	9% (*n* = 27)	9% (*n* = 26)	12% (*n* = 35)	7% (*n* = 23)	13% (*n* = 41)	12% (*n* = 36)
Growth	75% (*n* = 228)	85% (*n* = 259)	76% (*n* = 233)	89% (*n* = 270)	77% (*n* = 234)	82% (*n* = 249)

Percentages rounded to the nearest whole number.

#### 3.2.1 Language mindsets and their association with background variables

Next, the study investigated how the students’ language mindsets were stratified by various background variables. No statistically significant correlations were found between the participants’ language mindsets and their age, gender, or level of the language course. By contrast, a statistically significant difference was found between the language mindsets of Finns and non-Finns as measured by one of the six factors of the LMI: entity beliefs about an age-sensitive period. More specifically, a paired samples *t*-test showed that Finns were less fixed-minded about an age-sensitive period for language acquisition than their non-Finnish peers: *t*(304) = −2.046, *p* = 0.042 (Table 10).

**Table 10:** Mean scores for the six factors by nationality.

N 304	Mean Finn	SD	Mean other	SD
GLB-ent	2.6525	1.06521	2.6408	1.27719
GLB-inc	2.2589	0.88111	2.2529	0.92064
L2B-ent*	2.5727	0.96955	2.6695	1.10116
L2B-inc	2.2376	0.83822	2.1983	0.84554
ASB-ent*	**2.3848**	0.92516	**2.6236**	1.08350
ASB-int	2.2943	0.88206	2.4598	1.01358

^*^Items were reverse scored to allow easier comparison. Higher scores in the entity items signify a stronger fixed mindset; higher scores in the incremental items signify a weaker growth mindset. Means that differed to a statistically significant degree (*p* = <0.05) are in bold.

## 4 Discussion

In answer to RQ1a, “What are the psychometric properties of the Language Mindset Inventory in a Finnish higher education setting?” the present study replicated many of the findings of Lou and Noels (2017) with their original Canadian sample, such as the factor structure of the instrument.

### 4.1 LMI factor structure

When testing the construct validity of the scale with the Finnish data, CFA indicated that a six-factor model fit the data best, which was also the conclusion of Lou and Noels in their study (2017). In other words, the subscales were too distinct to reflect a single factor, language mindsets. Moreover, the entity (fixed) and incremental (growth) items within each subscale were too distinct to reflect a single factor for each subscale: general language beliefs, L2 beliefs, and age-sensitive beliefs.

The findings of the hierarchical factor analysis also mirrored those of Lou and Noels (2017), with the one-factor second-order model showing acceptable fit and the two-factor second-order a good fit. In their original article (2017), Lou and Noels argue that the CFA and HFA results justify three strategies for using the LMI: retaining the separateness of the six factors (based on the CFA results), creating a composite score for the three entity factors and the three incremental factors, and creating a single composite score for all six factors (based on the HFA results).

The results for the Finnish data suggest that the six-factor strategy might be the most appropriate. First, of all the models tested, the six-factor model fit the data best. Second, the correlations between the six factors indicated that the constructs may be too distinct to use composite scores. Indeed, in the Finnish data, some LMI factors were more closely associated with Dweck’s Implicit Theories of Intelligence scale than with other LMI factors (Tables 5, 7). This finding differs from Lou and Noels (2017), who found that the LMI factors were clearly more strongly correlated with each other than with Dweck’s scale. In turn, based on the current results, beliefs about innate giftedness seem to represent a more distant construct, less strongly correlated with both the LMI and implicit theories of intelligence, which provides support for the domain specificity of mindsets (e.g., O’Keefe et al., 2018).

### 4.2 Fixed and growth mindsets

Mirroring the findings of Lou and Noels (2017), the entity factors of the LMI tended to correlate more strongly with each other than with the incremental (growth) factors of the other subscales. Moreover, the entity and incremental factors within each subscale were only moderately correlated. Similar findings in their data led Lou and Noels (2017) to suggest that fixed and growth mindsets about languages were not two ends of a bipolar construct but rather distinct but related systems. For instance, they state that “This finding indicates that many people can be flexible and dialectical thinkers who ascribe to seemingly contradictory concepts if they are not forced to choose one concept or the other, and thus endorsing both entity and growth theories” (Lou and Noels, 2019, p. 534). This interpretation also provided their theoretical justification for creating a composite score for entity and growth language mindsets.

However, another possible explanation for these results is the effect of social desirability bias on the incremental (growth) items. In other words, some respondents might agree with growth-mindset items irrespective of their actual beliefs. That interpretation could be supported by the finding in the present study of a statistically significant difference between the mean scores for the growth mindset factors and the fixed mindset factors. More specifically, the respondents were more growth oriented as measured by the growth mindset factors than by the fixed mindset factors. This was also the case in Lou and Noels (2017).

In answer to RQ2, “What are the language mindsets of university students in Finland?” the data indicated that these students were growth minded as measured by all the six factors of the LMI. Lou and Noels (2017) do not provide individual mean scores for the six factors in their study, but they do list the combined means for the fixed and growth factors: 3.12 and 2.64 (reverse scored), respectively. In the present study, the mean scores for the three fixed-mindset factors ranged from 2.48 to 2.65, while the mean scores for the three growth-mindset factors varied from 2.24 to 2.36 (note a higher score = a more fixed mindset). Therefore, the students in the present study appeared to be more growth-mindset oriented than the students in Lou and Noels’ Canadian study. In addition, the difference in the students’ growth mindsets as measured by the fixed and growth items was smaller in the present study than in Lou and Noels (2017), although still statistically significant. One possible explanation for this finding is a stronger social desirability bias to agree with growth mindset items in a North American context, which some scholars have noted (e.g., Barger et al., 2022).

Moreover, the students in the Finnish study exhibited a stronger growth orientation about languages than they did about general intelligence, and this difference was also statistically significant. The finding is surprising given that a study by Ryan and Mercer (2012) found that Austrian university students held more of a fixed mindset about language learning than general intelligence. The same study nevertheless found that Japanese university students were more growth-oriented about languages than general intelligence, pointing to clear cultural differences. As measured by the entity factors of the LMI, between 10 and 16 percent of the participants in the present study were nonetheless fixed-minded about languages, the equivalent of five individuals in a class of 30 students. Thus, even in this this growth-oriented context, students might benefit from mindset interventions.

## 5 Conclusion

This study sought to evaluate the psychometric properties of the Language Mindsets Inventory (LMI) in a novel setting: Finnish higher education. In addition, it investigated the nature of university students’ language mindsets and how they differed from their general mindsets about intelligence and giftedness. The study replicated the findings of Lou and Noels on the factor structure of the LMI. However, in contrast to Lou and Noels, it found that some LMI factors were more closely correlated with Dweck’s ITI scale than with other LMI factors. The study also found that the higher education students in its sample tended to be growth minded about language learning and that this growth orientation was stronger than for beliefs about general intelligence and giftedness.

### 5.1 Implications

The study findings indicate that the LMI is a valid instrument for measuring the language mindsets of university students in Finland. However, the results also suggest that the constructs of general language beliefs, second language beliefs and age-sensitive beliefs are quite distinct. Therefore, creating composite scores may not be a valid strategy. Another approach suggested by and occasionally adopted by Lou et al. (2022) is to use just one subscale. The L2 beliefs subscale is most directly related to learning a second language and may provide the best information for second language educators. In this context, the other subscales might be, as Lou and Noels state, “tangential” to the research aims (2022, p. 8).

Another reason for using the L2B subscale alone could be parsimony. An 18-item instrument is rather unwieldy, particularly when mindsets are typically measured by three- or four-item scales. Respondent fatigue is a well-documented phenomenon and an obstacle to survey data collection (Ben-Nun, 2008). Nonetheless, even when using this subscale, it may be advisable to avoid calculating a composite mean score for the entity and incremental items due to their moderate correlations. This was the strategy adopted by Zarrinabadi et al. (2022a,b), where Nigel Lou was a coauthor.

### 5.2 Limitations

One limitation of the present study is the nature of the sample, which was heterogeneous, consisting of students from 48 countries. In a study attempting to test the cultural validity of a new instrument, this is a weakness. Nevertheless, the students were functioning in a Finnish educational environment reflecting Finnish educational values. Moreover, most respondents were Finnish nationals, and no statistically significant differences in language mindsets were found between the Finns and their non-Finnish peers, with the exception of one factor of the LMI, entity beliefs about an age-sensitive period for language learning.

A further limitation is that the study sample was drawn from one university in the Helsinki metropolitan area. Consequently, caution is required when generalizing the findings to other contexts.

Nonetheless, the present study represents an important contribution to the literature. It is the first study to investigate the psychometric properties of the LMI in a Nordic context, and it offers important insights into how the instrument should be used when comparing language mindsets to other variables. Moreover, the study is, to the authors’ knowledge, the first peer-reviewed research to examine the language mindsets of university students in Finland and their relationship to general mindsets about intelligence.

### 5.3 Future research

The study findings indicate several interesting directions for future research. First, for language mindset research and mindset research in general, it would be essential for future studies to determine the cause of the difference between mindsets as measured by entity items and mindsets as measured by incremental items. As mentioned earlier, the literature suggests four potential reasons for this: the distinctness of the growth and fixed mindset constructs, poor wording of the items, acquiescence, and social desirability bias. Of these, it seems unlikely that poor wording or acquiescence could account for growth mindsets being consistently stronger when measured by incremental items. However, the other two possibilities could be investigated by future studies using a mindset scale containing entity and growth items (such as the LMI) combined with qualitative participant interviews about the scale items and the participants’ beliefs about fixed and growth mindsets.

Regarding the three subscales of the LMI, it would also be important for future research to further explore how general language beliefs, L2 beliefs and age-sensitive beliefs form the basis of people’s mindsets about (primarily) second language acquisition. For instance, Lou and Noels (2017) base their inclusion of general language intelligence items on two studies by Mercer and Ryan (2010) and Ryan and Mercer (2012), but these studies do not explore this theme in great detail. Moreover, the wider literature remains ambivalent about the connection between native language and foreign language aptitude. Consequently, strengthening the theoretical underpinnings of the LMI would be an important task for future studies. In particular, there is a need for more studies demonstrating that beliefs about native language ability form a part of lay theories of second language acquisition.

## Data Availability

The raw data supporting the conclusions of this article will be made available by the authors, without undue reservation.
